# Permutation-Based Distances for Groups and Group-Valued Time Series

**DOI:** 10.3390/e27090913

**Published:** 2025-08-28

**Authors:** José M. Amigó, Roberto Dale

**Affiliations:** Centro de Investigación Operativa, Universidad Miguel Hernández, 03202 Elche, Spain; rdale@umh.es

**Keywords:** finite groups, permutations, ordinal patterns, transcripts, edit distance, Cayley and Kendall distances, Cayley’s theorem, algebraic representations, group-valued time series, time series analysis

## Abstract

Permutations on a set, endowed with function composition, build a group called a symmetric group. In addition to their algebraic structure, symmetric groups have two metrics that are of particular interest to us here: the Cayley distance and the Kendall tau distance. In fact, the aim of this paper is to introduce the concept of distance in a general finite group based on them. The main tool that we use to this end is Cayley’s theorem, which states that any finite group is isomorphic to a subgroup of a certain symmetric group. We also discuss the advantages and disadvantage of these permutation-based distances compared to the conventional generator-based distances in finite groups. The reason why we are interested in distances on groups is that finite groups appear in symbolic representations of time series, most notably in the so-called ordinal representations, whose symbols are precisely permutations, usually called ordinal patterns in that context. The natural extension from groups to group-valued time series is also discussed, as well as how such metric tools can be applied in time series analysis. Both theory and applications are illustrated with examples and numerical simulations.

## 1. Introduction

Symbolic representation of real-valued times series is a usual and useful tool in data analysis, where numbers are replaced by discrete “symbols”, in order to gain more tools and insights [1]. So to speak, symbolic representations coarse-grain the data in such a way that the information retained is sufficient for the purposes of the analysis. From a mathematical point of view, this technique consists of partitioning the state space, both in statistics and nonlinear methods. Traditional examples include binning and thresholding. More recently, Bandt and Pompe [2] proposed to use ordinal patterns, which are the rank vectors of sliding windows along a time series, the size of the windows being the length of the ordinal patterns. Since then, ordinal representations, i.e., symbolic representations with ordinal patterns, have become a popular technique among data analysts. Common applications of ordinal patterns include classification using ordinal pattern-based indices [3,4,5], discrimination of chaotic signals from white noise [6,7], characterization of dynamics and couplings [2,8,9] and nonparametric tests of serial dependence [10,11], to mention a few. For general overviews, see [12,13,14].

More importantly for the topic of this paper, ordinal patterns of any given length L≥2 can be interpreted as permutations (i.e., bijections) on any set of *L* elements, say, {1,2,…,L}. In fact, the Shannon entropy of a probability distribution of ordinal patterns is called permutation entropy [2], and the same happens with any other entropic functional based on ordinal pattern probability distributions, e.g., divergence, mutual information, or statistical complexity. A potential advantage of viewing ordinal patterns of length *L* as permutations is that the latter build a group, namely, the symmetric group of degree *L*, denoted by Sym(L), where the binary operation is function composition. In fact, the algebraic structure of Sym(L) provides additional leverage to ordinal representations that can be harnessed in time series analysis. An example of this is the concept of transcript introduced in [8].

More generally, symbolic representations whose symbols are elements of a group are called algebraic representations, an ordinal representation being an algebraic representation with alphabet Sym(L). Actually, most results for ordinal representations can be readily generalized to algebraic representations whose alphabets are any other finite group G. This is not surprising if no particular property of Sym(L) is used in a given proof or application. There may be another, more theoretical reason for this. According to Cayley’s theorem [15], any finite group G is isomorphic to a subgroup of a symmetric group. This means that permutations are a sort of universal symbol for discretizing time series by means of group elements; a different question is whether such a “canonical” embedding is always the best option in practice.

This being the case, in this paper, we extend two distances in Sym(L), namely, the Cayley distance and the Kendall tau distance (henceforth called Kendall distance), to arbitrary finite groups via Cayley’s theorem. A possible advantage of the here-proposed distances compared to others (e.g., the conventional generator-based distances) is their expediency and acceptable computation time for groups of moderate cardinality, as happens in practice. By extension, we discuss also distances for group-valued time series, which include algebraic representations of time series. This issue raises naturally when comparing two time series to measure their “similarity” (think of classification or clustering) or studying coupled systems (think of different types of synchronization). The result is a suite of permutation-based (or ordinal pattern-based) distances for groups and group-valued time series.

In sum, this is a follow-up paper on the quest to exploit the algebraic structure of group-valued time series—a possibility rarely used in the literature. Remarkably, the Cayley and Kendall distances and, hence, their extensions to general groups, are actually norms of transcripts, which shows the potential of our algebraic approach. Since our interest in distances between group elements was motivated by the study of ordinal representations and transcripts, we will speak of both permutations and ordinal patterns.

To address the aforementioned topics, we begin in Section 2 by establishing the mathematical framework, which includes group actions and group representations. In particular, we will prove Cayley’s theorem and implement it in three different ways—one of them using transcripts. There and throughout this paper, our approach is formal, the theoretical concepts being illustrated with simple examples. Section 3 is dedicated to the symmetric group and its two standard metrics: the Cayley and Kendall distances. In Section 4, we transition from the symmetric group to general groups and propose a distance based on Cayley’s Theorem (Section 4.1). This distance is compared to the conventional string metric for finitely generated groups (Section 4.2) in Section 4.3. Possible extensions to distances between group-valued times series are discussed in Section 5 and illustrated with mathematical simulations in Section 6. This paper ends with the conclusions in Section 7.

## 2. Groups, Group Actions and Cayley’s Theorem

In this section, we set the mathematical framework of this paper—group actions and group representations [15,16,17].

**Definition** **1.** A group (G,∗) is a nonempty set G endowed with a binary operation “*", sometimes called composition law or product, satisfying the following properties.

**(G1)** 
Associativity: *For all a,b,c∈G, it is true that (a∗b)∗c=a∗(b∗c).*
**(G2)** 
Identity element: *There exists an element e∈G, called the identity (or neutral) element, such that a∗e=e∗a=a for all a∈G.*
**(G3)** 
Inverse element: *For every a∈G, there exists an element $a^{-1}\in G$, called the inverse element of a, such that $a*a^{-1}=a^{-1}*a=e$.*

It can be proved that the identity of a group and the inverse of each element are unique. Groups whose product is commutative (i.e., a∗b=b∗a for all a,b∈G) are called *commutative* or *abelian*. Examples of abelian groups are the real numbers endowed with addition and the nonzero real numbers endowed with multiplication. Invertible square matrices are examples of nonabelian groups under multiplication. If the binary operation is clear from the context, then (G,∗) is shortened to G.

**Definition** **2.** *If (G,∗) is a group and S a nonempty set, then a left group action of G on S is a mapping F:G×S→S such that it satisfies the following two axioms:*

**(L1a)** 
Identity: *F(e,s)=s for all s∈S, where e is the identity element of G.*
**(L2a)** 
Compatibility: *F(a,F(b,s))=F(a∗b,s) for all a,b∈G and s∈S.*

If *F* is a left action of G on *S*, we can define the function $F_{a}:=F\left(a,\cdot \right):S\to S$, i.e.,

(1) $$F_{a}\left(s\right)=F\left(a,s\right)$$

for each a∈G, for $F_{a}$, the axioms L1a and L2a read as follows:

**(L1b)**  Identity: *$F_{e}$ is the identity mapping s↦s for all s∈S.*
**(L2b)** 
Compatibility: *$F_{a}\circ F_{b}=F_{a*b}$ for all a,b∈G.*

**Lemma** **1.** *(i)* *$F_{a}:S\to S$ is a bijection for each a∈G.*

*(ii)*  *The set $\left\{F_{a}:S\to S:a\in G\right\}$ endowed with function composition is a group.*

**Proof.**  (i) Since $F_{a}$ is defined from *S* into itself, it suffices to prove that every s∈S has an inverse. Indeed, $F_{a}^{-1}\left(s\right)=F_{a^{-1}}\left(s\right)\in S$ because $F_{a}\left(F_{a^{-1}}\left(s\right)\right)=F_{a*a^{-1}}\left(s\right)=F_{e}\left(s\right)=s$ by axioms L2b and L1b.

(ii) According to Definition 1, we have to prove three properties: (G1) associativity is a general property of the composition of functions; (G2) $F_{e}$ is the identity because of axiom L1b; (G3) for all mappings $F_{a}$, ${\left(F_{a}\right)}^{-1}=F_{a^{-1}}$ as in (i).

□

Bijections from a finite set *S* onto itself are called *permutations*. So, according to Lemma 1*(i)*, the mappings $F_{a}$ are permutations. The permutations on *S*, endowed with function composition, build a group called the *symmetric group* Sym(S). In this paper, we consider only finite groups G and finite sets *S*, so, if $\left|S\right|$ is the cardinality of *S*, then $\left|\mathrm{Sym}(S)\right|=\left|S\right|!$. Since the properties of the permutations on *S* do not depend on *S* but only on $\left|S\right|$, we choose $S=\{1,2,\ldots ,\left|S\right|\}$, unless otherwise stated, and also refer to Sym(S) as the symmetric group of degree $\left|S\right|$, $\mathrm{Sym}(\left|S\right|)$. As a historical note, the symmetric group goes back to Évariste Galois (1811–1832) and his work on the resolution of algebraic equations by means of radicals.

Furthermore, Lemma 1*(ii)* states that the set of permutations $\left\{F_{a}:S\to S:a\in G\right\}$ is a subgroup (of cardinality $\left|G\right|$) of Sym(S). This result together with axiom L2b, which spells out that the mapping $\Phi :a\mapsto F_{a}$ preserves the algebraic structure of G, are merged in the following theorem.

**Theorem** **1.** *Any (left) group action F:G×S→S of a group G on a finite set S defines a group homomorphism $\Phi :a\mapsto F_{a}$ from G into Sym(S). Therefore,* Φ *is a representation of the group G by means of permutations $F_{a}:=F\left(a,\cdot \right):S\to S$.*

In other words, every group G is isomorphic to a subgroup H of Sym(S), namely H=Φ(G), hence, $\left|H\right|=\left|G\right|$. In this formulation, Theorem 1 is known as *Cayley’s theorem*. Therefore, we will call Φ:G→Sym(S) Cayley’s homomorphism and, abusing notation, Φ:G→H Cayley’s isomorphism. Below, we will discuss three different implementations of Cayley’s isomorphism.

To apply Theorem 1, label the elements of G with the conventional set $\left\{1,2,\ldots ,\left|G\right|\right\}$. For every a∈G, let

(2) $$F_{a}=\left(\begin{array}{ccccc} 1 & \ldots & k & \ldots & \left|G\right|\\ F_{a}\left(1\right) & \ldots & F_{a}\left(k\right) & \ldots & F_{a}\left(\left|G\right|\right) \end{array}\right)=\left(\begin{array}{ccccc} 1 & \ldots & k & \ldots & \left|G\right|\\ n_{1} & \ldots & n_{k} & \ldots & n_{\left|G\right|} \end{array}\right)$$

be the matrix (or two-line) form of the permutation $F_{a}$, where $\left(n_{1},\ldots ,n_{k},\ldots ,n_{\left|G\right|}\right)$ is a shuffle of $\left(1,2,\ldots ,\left|G\right|\right)$. Therefore, every element a∈G can be identified with the *one-line form* $\left(n_{1},n_{2},\ldots ,n_{\left|G\right|}\right)$ of $F_{a}$. In the numerical examples below, we will juxtapose the components of $\left(n_{1},n_{2},\ldots ,n_{\left|G\right|}\right)$ and drop the parentheses for a compact notation.

**Remark** **1.** *In addition to left actions of a group (G,∗) on a finite set S, there are also right actions $\tilde{F}:S\times G\to S$, defined by (R1a) $\tilde{F}\left(s,e\right)=s$ for all s∈S, and (R2a) $\tilde{F}\left(\tilde{F}\left(s,a\right),b\right)=\tilde{F}\left(s,a*b\right)$, as well as the corresponding group homomorphism $a\to {\tilde{F}}_{a}:=\tilde{F}\left(\cdot ,a\right)$ from G to Sym(S), such that (R1b) ${\tilde{F}}_{e}$ is the identity map s↦s for all s∈S, and (R2b) ${\tilde{F}}_{a}\circ {\tilde{F}}_{b}={\tilde{F}}_{a*b}$ for all a,b∈G. The difference between left and right actions is that in the function composition $F_{a}\circ F_{b}=F_{a*b}$ (L2b), $F_{b}$ acts first on s∈S and $F_{a}$ second (as in the standard convention), whereas in ${\tilde{F}}_{a}\circ {\tilde{F}}_{b}={\tilde{F}}_{a*b}$ (R2b), ${\tilde{F}}_{a}$ acts first on s∈S and ${\tilde{F}}_{b}$ second. Henceforth, we only consider left actions because the binary operation of the symmetric group, the main character of this paper, is precisely function composition and so we can use the standard convention.*

There is a particular case of Theorem 1 that is of special interest here, namely, S=G, i.e., when the group G acts on itself. In this particular case, we are going to highlight three implementations of Cayley’s isomorphism $\Phi :G∋a\mapsto F_{a}\in \mathrm{Sym}\left(G\right)$ via left actions.

**(A)** 
*Left translations*: The mapping (a,b)↦Λ(a,b)=a∗b is a left action of G on itself, so
  (3) $$\Lambda_{a}\left(b\right)=a*b$$
  is a permutation on G for every a∈G, called a left translation by *a*.
**(B)** 
*Right translations*: The mapping $\left(a,b\right)\mapsto R\left(a,b\right)=b*a^{-1}$ is a left action of G on itself, so
  (4) $$R_{a}\left(b\right)=b*a^{-1}$$
  is a permutation on G for every a∈G, called a right translation by *a*. Let us mention that the operation R(a,b) is also called the *transcription* from the (source) symbol *a* to the (target) symbol *b* in [8]. Note that $\Lambda_{a}\left(b\right)=R_{b^{-1}}\left(a\right)$ and $R_{a}\left(b\right)=\Lambda_{b}\left(a^{-1}\right)$.
**(C)** 
*Adjoint actions*: The mapping $\left(a,b\right)\mapsto \mathrm{Ad}\left(a,b\right)=a*b*a^{-1}$ is a left action of G on itself, so
  (5) $${\mathrm{Ad}}_{a}\left(b\right)=a*b*a^{-1}$$
  is a permutation on G for every a∈G, called the adjoint action of *a*.

Comparing Equations (3)–(5), we conclude that the implementation (3) of Cayley’s isomorphism $\Phi :a\mapsto F_{a}$ is the most convenient in practice, since the (one-line form of the) permutations $\Lambda_{a}:b\mapsto a*b$ can be read immediately row by row in the multiplication table of G. Indeed, if $\left\{a_{1},a_{2},\ldots .,a_{\left|G\right|}\right\}$ is an enumeration of the elements of G, then $\Lambda_{a_{i}}$ is the *i*-th row of the multiplication table ${\left(a_{i}*a_{j}\right)}_{1\leq i,j\leq \left|G\right|}$, i.e.,

(6) $$\Lambda_{a_{i}}=\left(a_{i}*a_{1},\ldots ,a_{i}*a_{j},\ldots ,a_{i}*a_{\left|G\right|}\right)=\left(\begin{array}{ccccc} a_{1} & \ldots & a_{j} & \ldots & a_{\left|G\right|}\\ a_{i}*a_{1} & \ldots & a_{i}*a_{j} & \ldots & a_{i}*a_{\left|G\right|} \end{array}\right).$$

**Example** **1.** *Let G=Sym(3). By Equation (3), the isomorphic copies $\Lambda_{r}\in \mathrm{Sym}\left(G\right)=\mathrm{Sym}\left(\mathrm{Sym}\left(3\right)\right)=\mathrm{Sym}\left(6\right)$ of r∈{123,132,213,231,312,321} are given by the rows of the “multiplication" table of Sym(3),*

(7)

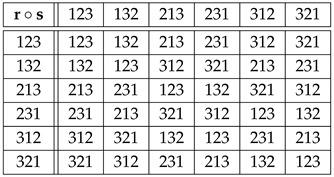


*where r∘s stands for the composition of the permutation r that labels a row with the permutation s that labels a column. Therefore,*

(8)

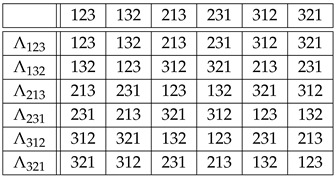


*For example,*

$$\Lambda_{231}:123\mapsto 231,\;132\mapsto 213,\;213\mapsto 321,\;231\mapsto 312,\;312\mapsto 123,\;321\mapsto 132,$$

*or, in one-line form, $\Lambda_{231}=\left(231,213,321,312,123,132\right)$. From*

$$123^{-1}=123,\;132^{-1}=132,\;213^{-1}=213,\;231^{-1}=312,\;312^{-1}=231,\;321^{-1}=321,$$

*table (7) and Equation (4), we obtain similarly that the copies $R_{r}\in \mathrm{Sym}\left(\mathrm{Sym}\left(3\right)\right)$ of r∈Sym(3) via right translations are given by*

(9)

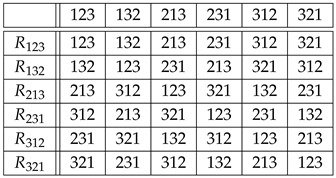

**Example** **2.** *Let $G=\{\theta^{0},\theta^{1},\theta^{2},\theta^{3}\}$ endowed with the product $\theta^{i}*\theta^{j}=\theta^{j}*\theta^{i}=\theta^{i+j}$ where, in this example, the exponents are taken modulo* 4. *Hence, $\theta^{0}$ is the identity and ${\left(\theta^{i}\right)}^{-1}=\theta^{4-i}$. By definition, G is a cyclic group generated by the element $\theta^{1}$. Alternatively, G can be identified with the additive group {0,1,2,3}, where the sum is taken modulo* 4.

*(i) The four permutations $\Lambda_{\theta^{i}}:\theta^{j}\mapsto \theta^{i}*\theta^{j}=\theta^{i+j}$, corresponding to Equation (3) under the isomorphism $\Phi :\theta^{i}\mapsto$$\Lambda_{\theta^{i}}\in \mathrm{Sym}\left(G\right)$, are given in the following table:*

(10)

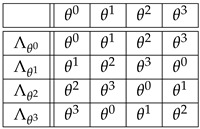


*So, for instance, the second row of this table spells out*

$$\Lambda_{\theta^{1}}:\theta^{0}\mapsto \theta^{1},\;\theta^{1}\mapsto \theta^{2},\;\theta^{2}\mapsto \theta^{3},\;\theta^{3}\mapsto \theta^{0},\;$$

*or $\Lambda_{\theta^{1}}=\left(\theta^{1},\theta^{2},\theta^{3},\theta^{0}\right)$.*

*(ii) The four permutations $R_{\theta^{i}}:\theta^{j}\mapsto \theta^{j}*{\left(\theta^{i}\right)}^{-1}=\theta^{j-i}$, corresponding to Equation (4), under the isomorphism $\Phi :\theta^{i}\mapsto$$R_{\theta^{i}}\in \mathrm{Sym}\left(G\right)$, are given in the following table:*

(11)

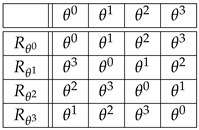


*So, if in table (10), $\Lambda_{\theta^{i+1}}$ is obtained from $\Lambda_{\theta^{i}}$ by a clockwise (negative) circular shift, in table (11), the circular shift to obtain $R_{\theta^{i+1}}$ from $R_{\theta^{i}}$ is counterclockwise (positive).*

## 3. Ordinal Patterns and Distances

In the previous, section we have focused on group actions and the embedding of a group in a symmetric group. What is still missing is metric tools that can further boost applications in the realm of group-valued time series. Since the motivation and objective of this paper are the applications of such tools to symbolic representations of time series via group elements, we begin this section by briefly explaining how such symbolic representations arise in time series analysis. The choice of ordinal patterns (or permutations) responds to the popularity of these symbols among time series analysts. Then, we introduce the concept of distance in the symmetric group and, in the next section, we do the same for general groups.

### 3.1. Ordinal Patterns

Symmetric groups are very popular for symbolic representations since the concept of *ordinal pattern* was introduced in [2]. Given a real-valued time series $x={\left(x_{t}\right)}_{t\geq 0}$, an *ordinal representation* of *x* is a symbolic time series ${\left(r_{t}\right)}_{t\geq 0}$ whose alphabet is Sym(L), the symmetric group of degree L≥2. How are the permutations $r_{t}$ obtained from *x*? Let $x_{t}^{L}:=x_{t},x_{t+1},\ldots ,x_{t+L-1}$ be a window (segment, sequence, block, …) of size *L*. Then, $r_{t}=\left(r_{1},r_{2},\ldots ,r_{L}\right)$ is the rank vector of $x_{t}^{L}$, that is, $\left(r_{1},r_{2},\ldots ,r_{L}\right)$ is the permutation of {1,2,…,L} such that

(12) $$x_{t+r_{1}-1}<x_{t+r_{2}-1}<\ldots <x_{t+r_{L}-1}.$$

In other words, the rank vector $r_{t}$ is viewed as the one-line form of the permutation $1\mapsto r_{1}$, $2\mapsto r_{2}$, …, $L\mapsto r_{L}$, i.e., $r_{t}\left(k\right)=r_{k}$ for 1≤k≤L. As a matter of fact, any total ranking can be viewed as a permutation. In case of a tie $x_{i}=x_{j}$, one can apply the convention that $x_{i}<x_{j}$ if i<j. Another possibility, more recommended in case of many ties, is to add a small-amplitude noise to $x_{i}$ and $x_{j}$ to undo the tie. As way of illustration, if L=4 and $x_{t}^{L}=2.1,\;0.3,\;1.5,\;2.4$, then $r_{t}=\left(2,3,1,4\right)$, or $r_{t}=2314$ for short.

In [2], the permutations $r_{t}$ were called order (or ordinal) patterns of length *L*, which is the usual name of the symbols $r_{t}$ in time series analysis. In addition to the length *L* of the patterns, ordinal representations depend also on a second parameter: a possible time delay in Equation (12). In this paper, the time delay is set equal to 1 throughout.

As a side note, the concept of ordinal pattern has been generalized in several directions. Thus, it has been extended to multivariate time series in [18,19]. Spatial ordinal patterns were introduced in [20] to analyze two-dimensional images and applied in [21,22] to distinguish textures.

### 3.2. Distances for Ordinal Patterns

In this section, we introduce the Cayley and Kendall distances for the symmetric group Sym(L); see [23] for a survey about distances on permutations. We remind first about the concept of distance.

**Definition** **3.** *Given a nonempty set S, a distance is a function d:S×S→R that satisfies the following three axioms for all points x,y,z∈S.*

**(D1)** 
Positivity: *d(x,y>0 and d(x,y)=0 if and only if x=y.*
**(D2)** 
Symmetry: *d(x,y)=d(y,x).*
**(D3)** 
Triangular inequality: *d(x,z)≤d(x,y)+d(y,z).*

Following the notation in Section 3.1 for ordinal patterns, the permutations of Sym(L) will be written in the one-line form $r=(r_{1},r_{2},\ldots ,r_{L})$ (possibly shortened to $r_{1},r_{2},\ldots ,r_{L}$ in numerical examples), where $r\left(i\right)=r_{i}$. If, furthermore, $s=\left(s_{1},s_{2},\ldots ,s_{L}\right)\in \mathrm{Sym}\left(L\right)$, then r∘s is the usual function composition (r∘s)(i)=r(s(i)), i.e.,

(13) $$r\circ s=\left(r_{1},\ldots ,r_{k},\ldots ,r_{L}\right)\circ \left(s_{1},\ldots ,s_{k},\ldots ,s_{L}\right)=\left(r_{s_{1}},\ldots ,r_{s_{k}},\ldots ,r_{s_{L}}\right),$$

as exemplified in Equation (7) for L=3. Due to the positivity and symmetry properties of a distance, the L!×L! *distance matrix* (d(r,s):r,s∈Sym(L)) is symmetric, with 0’s along the diagonal.

If $\left\{i_{1},i_{2},\ldots ,i_{m}\right\}\subset \left\{1,2,\ldots ,L\right\}$, then $\left(i_{1},i_{2},\ldots ,i_{m}\right)$ denotes the permutation

(14) $$i_{1}\mapsto i_{2},\;i_{2}\mapsto i_{3},\;\ldots ,\;i_{m-1}\mapsto i_{m},\;i_{m}\mapsto i_{1},\;$$

called a *cycle of length m*, 1≤m≤L, or simply an *m*-cycle. The notation calls for a warning at this point: do not confuse the permutation $i_{1},i_{2}\ldots ,i_{m}=\left(i_{1},i_{2},\ldots ,i_{m}\right)$ with the cycle $\left(i_{1},i_{2},\ldots ,i_{m}\right)$. Every permutation can be written as a product of disjoint cycles, which is unique except for the order of the factors. For example, the cycle factorization of the permutation 426135 is (14)(2)(356) or (14)(356) if 1-cycles (“fixed elements”) are omitted.

Cycles of length 2 are called *transpositions*. That is, a transposition is a permutation $t_{ij}\in \mathrm{Sym}\left(L\right)$ such that $t_{ij}\left(i\right)=j$, $t_{ij}\left(j\right)=i$, and $t_{ij}\left(k\right)=k$ for all k≠i,j. If $r=(r_{1},\ldots ,r_{L})$, then

(15) $$r\circ t_{ij}=\left(r_{1},\ldots ,r_{i-1},r_{j},r_{i+1},\ldots ,r_{j-1},r_{i},r_{j+1},\ldots ,r_{L}\right).$$

If $\left|i-j\right|=1$, then $t_{ij}$ is called an *adjacent transposition*. Unlike the factorization of permutations into disjoint cycles, the factorization of permutations into adjacent transpositions (and, hence, into transpositions) is not unique, although the minimal number of factors is. For example, 321=(12)(23)(12)=(23)(12)(23).

**Definition** **4** ([24,25])**.** *Let r,s∈Sym(L). (a) The* Cayley distance *between the two permutations r and s, denoted by $d_{C}\left(r,s\right)$, is defined as the minimum number of transpositions needed to transform r into s. (b) The* Kendall distance *(also known as the bubble-sort distance) between r and s, denoted by $d_{K}\left(r,s\right)$, is defined as the minimum number of* adjacent *transpositions needed to transform r into s.*

The Cayley and Kendall distances are examples of edit distances between two strings of symbols, which measure the minimum cost sequence of allowed edit operations to transform one string into the other. The use of edit distances to measure distance between permutations was proposed in [26]. By definition,

(16) $$d_{C}\left(r,s\right)\leq d_{K}\left(r,s\right)$$

for all r,s∈Sym(L).

The proofs of the positivity and symmetry (properties (D1) and (D2) in Definition 3) for $d_{C}\left(r,s\right)$ and $d_{K}\left(r,s\right)$ are straightforward. The triangular inequality can be easily proved by graph-based methods since the permutations of Sym(L) build a connected undirected graph where the nodes (or vertices) correspond to permutations and the links (or edges) to transpositions. For example, in the case of $d_{K}\left(r,s\right)$: (i) every node r is connected to exactly L−1 nearest neighbors, namely, those permutations that differ from r due to transpositions of the adjacent symbols $r_{i},r_{i+1}$ for 1≤i≤L−1, and, hence, (ii) for any two nearest nodes u and v, $d_{K}\left(u,v\right)=d_{K}\left(v,u\right)=1$. Therefore, $d_{K}\left(r,s\right)$ counts the number of links of the shortest path connecting the nodes r and s. In other words, each node has degree L−1 and all its nearest neighbors (one link apart) are at distance 1. The diameter of the graph, i.e., the farthest distance between any two nodes, corresponds to $r=(r_{1},r_{2},\ldots ,r_{L})$ and the order reversing permutation $s=(r_{L},r_{L-1},\ldots ,r_{1})$, hence

(17) $$d_{K,\max}\left(L\right)=\left(L-1\right)+\left(L-2\right)+\ldots +1=\frac{L(L-1)}{2}.$$

Such graphs are called *adjacency graphs* or networks.

**Figure 1:**
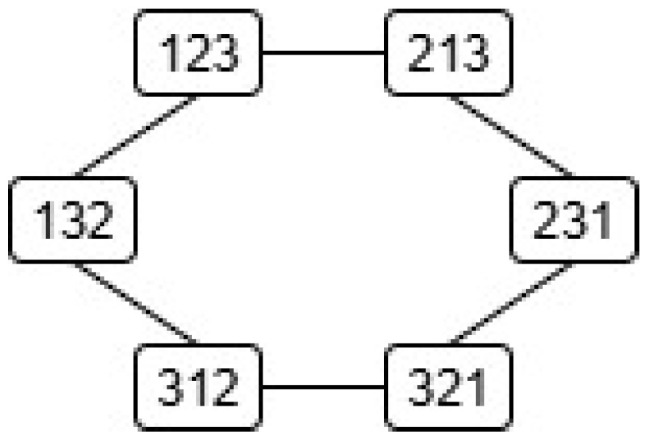
Kendall adjacency graph of Sym(3). A link between two nodes means that the corresponding permutations differ by an adjacent transposition, i.e., the Kendall distance between them is 1.

**Figure 2:**
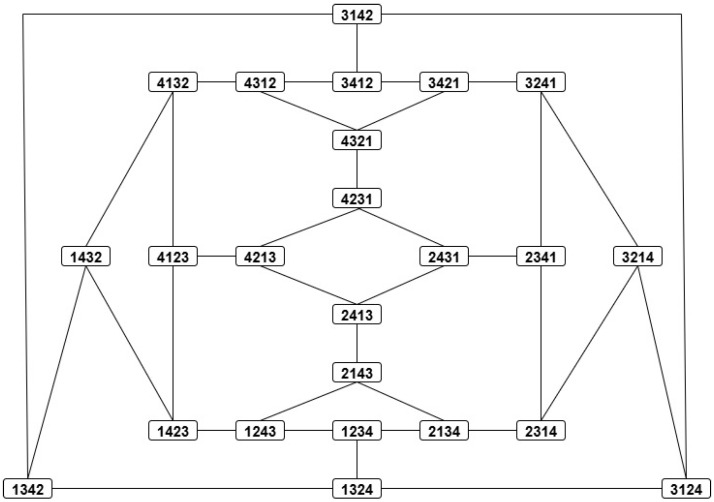
Kendall adjacency graph of Sym(4). A link between two permutations means that the Kendall distance between them is 1.

Figure 1 and Figure 2 show the adjacency graphs of the groups Sym(3) (a cycle in this case) and Sym(4), respectively. Unlike the adjacency graphs for the Kendall distance, the adjacency graphs for the Cayley distance are in general nonplanar, i.e., they have edge crossings (even for Sym(3)), so we will not use them.

In the following, whenever convenient for economy of notation, we denote by $d_{C,K}$ both the Cayley and Kendall distances.

**Proposition** **1** (Invariance of $d_{C,K}$ under left translations)**.** *Given r,s∈Sym(L), then*

(18) $$d_{C,K}\left(r,s\right)=d_{C,K}\left(u\circ r,u\circ s\right)$$

*for all u∈Sym(L).*

**Proof.**  Suppose $d_{C,K}\left(r,s\right)=k$, i.e., *k* is the mimimum number of transpositions or adjacent transpositions $t_{i_{1}j_{1}},t_{i_{2}j_{2}},\ldots ,t_{i_{k}j_{k}}\in \mathrm{Sym}\left(L\right)$ such that

$$r=\left(\ldots \left(\left(s\circ t_{i_{1}j_{1}}\right)\circ t_{i_{2}j_{2}}\right)\circ \ldots \circ t_{i_{k-1}j_{k-1}}\right)\circ t_{i_{k}j_{k}},$$

see Equation (15). Then,

$$u\circ r=\left(\ldots \left(\left(u\circ s\circ t_{i_{1}j_{1}}\right)\circ t_{i_{2}j_{2}}\right)\circ \ldots \circ t_{i_{k-1}j_{k-1}}\right)\circ t_{i_{k}j_{k}},$$

which proves that $d_{C,K}\left(u\circ r,u\circ s\right)=k.$ □

Since $d_{C,K}\left(r,s\right)=d_{C,K}\left(s,r\right)$, then $d_{C,K}\left(u\circ r,u\circ s\right)=d_{C,K}\left(u\circ s,u\circ r\right).$ Choose $u=r^{-1}$ or $u=s^{-1}$ in Equation (18) to prove:

**Corollary** **1.** *For every r,s∈Sym(L),*

(19) $$d_{C,K}\left(r,s\right)=d_{C,K}\left(e,r^{-1}\circ s\right)=d_{C,K}\left(e,s^{-1}\circ r\right),$$

*where e is the identity permutation.*

**Remark** **2.** *Owing to Equation (19), all possible values of $d_{C,K}\left(r,s\right)$ appear on the row $\left(d_{C,K}\left(e,u\right):u\in \mathrm{Sym}\left(L\right)\right)$ of the distance matrix.*

Equation (19) allows to define in Sym(L) an analogue to the concept of norm in a vector space.

**Definition** **5.** *The norm ${∥\cdot ∥}_{C,K}$ of r∈Sym(L) is defined as*

(20) $${∥r∥}_{C,K}=d_{C,K}\left(e,r\right).$$

Then, by Equation (19),

(21) $$d_{C,K}\left(r,s\right)={∥r^{-1}\circ s∥}_{C,K}={∥s^{-1}\circ r∥}_{C,K}.$$

**Remark** **3.** *The right translation of b∈G by a∈G, or the transcript from (the source) a to (the target) b, was defined in Equation (4) as $R\left(a,b\right)=b*a^{-1}$. In view of Equation (21), we conclude that the distance $d_{C,K}\left(r,s\right)$ is the norm ${∥\cdot ∥}_{C,K}$ of the right translations or transcripts $R\left(s^{-1},r^{-1}\right)=r^{-1}\circ s$ and $\;R\left(r^{-1},s^{-1}\right)=s^{-1}\circ r=$$R{\left(s^{-1},r^{-1}\right)}^{-1}$.*

Corollary 1 is instrumental for the computation of the Cayley and Kendall distances [24].

**Proposition** **2.** *(a) Let $u=\left(u_{1},\ldots ,u_{L}\right)\in \mathrm{Sym}\left(L\right)$ and C(u) the number of cycles (including 1-cycles) in the cycle factorization of the permutation u. Then,*

(22) $$d_{C}\left(r,s\right)=L-C\left(r^{-1}\circ s\right)=L-C\left(s^{-1}\circ r\right)$$

*for all r,s∈Sym(L)*

*(b) Let I(u) be the number of inversions in the permutation u, i.e., the number of ordered pairs $\left(u_{i},u_{j}\right)$, 1≤i<j≤L, such that $u_{i}>u_{j}$. Then,*

(23) $$d_{K}\left(r,s\right)=I\left(r^{-1}\circ s\right)=I\left(s^{-1}\circ r\right)$$

*for all r,s∈Sym(L).*

From Equation (22), it follows

(24) $$d_{C}\left(r,s\right)\in \left\{0,1,\ldots ,d_{C,\max}\left(L\right)\right\},\;\mathrm{where}\;d_{C,\max}\left(L\right)=L-1,$$

and, according to Equation (17),

(25) $$d_{K}\left(r,s\right)\in \left\{0,1,\ldots ,d_{K,\max}\left(L\right)\right\},\;\mathrm{where}\;d_{K,\max}\left(L\right)=\frac{L(L-1)}{2}.$$

**Example** **3.** *We illustrate Proposition 2 with L=6, r=462531 and s=236514. Then,*

$$s^{-1}={\left(\begin{array}{cccccc} 1 & 2 & 3 & 4 & 5 & 6\\ 2 & 3 & 6 & 5 & 1 & 4 \end{array}\right)}^{-1}=\left(\begin{array}{cccccc} 1 & 2 & 3 & 4 & 5 & 6\\ 5 & 1 & 2 & 6 & 4 & 3 \end{array}\right),$$

*so that*

$$s^{-1}\circ r=512643\circ 462531=631425,$$

*whose cycle factorization is*

$$s^{-1}\circ r=\left(16523\right)\left(4\right).$$

*According to Equation (22),*

(26) $$d_{C}\left(r,s\right)=L-C\left(s^{-1}\circ r\right)=6-2=4.$$

*As for Equation (23), the inversions of $s^{-1}\circ r$ are*

$$\begin{array}{ccc} & & (6,3),(6,1),(6,4),(6,2),(6,5),\\ & & (3,1),(3,2),\\ & & (4,2), \end{array}$$

*so that,*

(27) $$d_{K}\left(r,s\right)=I\left(s^{-1}\circ r\right)=8.$$

*Let us check the results (26) and (27). First, the transpositions needed to transform r into s are the following:*

$$\begin{array}{ccccc} r=462531 & \overset{\left(13\right)}{⟶} & 264531 & \overset{\left(25\right)}{⟶} & 234561\\ \; & \overset{\left(35\right)}{⟶} & 236541 & \overset{\left(56\right)}{⟶} & 236514=s \end{array}$$

*where the elements being swapped in each transposition have been boldfaced. Therefore, $d_{C}\left(r,s\right)=4$. To check Equation (27), call $\delta_{1}$ the number of adjacent transpositions needed to move in r the symbol 2 (the first or leftmost symbol of the target s) to the first position; call $r^{\left(1\right)}$ the result. Similarly, call $\delta_{2}$ the number of adjacent transpositions needed to move in $r^{\left(1\right)}$ the symbol 3 (the second symbol of the target s) to the second position. Proceed analogously until $r^{\left(k\right)}=s$. The adjacent transpositions needed to transform r into s in this example are the following:*

$$\begin{array}{ccccccc} r=462531 & \overset{\delta_{1}=2}{⟶} & r^{\left(1\right)}=246531 & \overset{\delta_{2}=3}{⟶} & r^{\left(2\right)}=234651 & \; & \\ \; & \overset{\delta_{3}=1}{⟶} & r^{\left(3\right)}=236451 & \overset{\delta_{4}=1}{⟶} & r^{\left(4\right)}=236541 & \overset{\delta_{5}=1}{⟶} & r^{\left(5\right)}=236514=s \end{array}$$

*where the element of $r^{\left(i\right)}$ ($r^{\left(0\right)}:=r$) being moved to the (i+1)-position has been boldfaced. This shows that $d_{K}\left(r,s\right)=\delta_{1}+\ldots +\delta_{5}=8$.*

**Example** **4.** *According to [27], G=Sym(3) is the most common ordinal representation in data analysis. The Cayley and Kendall distance matrices for the group Sym(3), Equation (7), are shown in the tables*

(28)

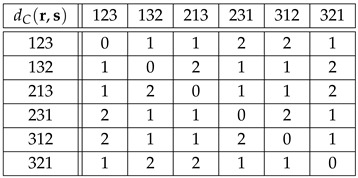


*and*

(29)

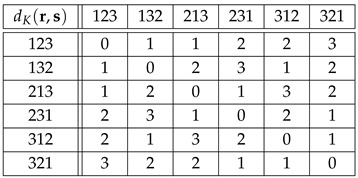


*As shown in Equations (16), (24) and (25), $d_{C}\left(r,s\right)\leq d_{K}\left(r,s\right)$ for all r,s∈Sym(3), $d_{C}\left(r,s\right)\in \left\{0,1,2\right\}$ and $d_{K}\left(r,s\right)\in \left\{0,1,2,3\right\}$.*

*Owing to their large size, the Cayley and Kendall distance matrices for G=Sym(4) have been moved to Appendix A. In this case, $d_{C}\left(r,s\right)\in \left\{0,1,2,3\right\}$, and $d_{K}\left(r,s\right)\in \left\{0,1,2,3,4,5,6\right\}$. Needless to say, the distances $d_{K}\left(r,s\right)$ in table (29) and Table A2 can be easily checked in the corresponding adjacency graphs, Figure 1 and Figure 2, where each link stands for distance 1.*

## 4. Distances for General Groups

In the first part of this section, we harness Cayley’s theorem to transport the Cayley and Kendall distances in Sym(L) (or, for that matter, any distance defined in Sym(L)) to any finite group (G,∗) with $\left|G\right|=L$. In the second part, we briefly introduce the distance with respect to a generating system. We also discuss the advantages of the first approach as compared to the second.

### 4.1. Permutation-Based Distance for Groups

Let Φ:G→H be Cayley’s isomorphism, where H is a subgroup of Sym(G) (namely, H=Φ(G)) with $\left|H\right|=\left|G\right|$). This means:

**(i)** 
Φ(e)$=(1,2,\ldots ,\left|G\right|)$, where *e* is the identity of G.
**(ii)** 
Φ(a∗b)=Φ(a)∘Φ(b) for all a,b∈G. Hence, $\Phi \left(a^{-1}\right)=\Phi {\left(a\right)}^{-1}$.

To endow G with a distance, we transport the distance $d_{C,K}\left(r,s\right)$ from the group Φ(G)⊂Sym(G) to G and promote Φ to an isometry.

**Definition** **6.** *Let* Φ *be the Cayley isomorphism for a finite group G. Then, $D_{C,K}^{\left(\Phi \right)}$ is the distance in G defined as*

(30) $$D_{C,K}^{\left(\Phi \right)}\left(a,b\right)=d_{C,K}\left(\Phi \left(a\right),\Phi \left(b\right)\right).$$

Therefore, $D_{C,K}^{\left(\Phi \right)}$ has the same properties as $d_{C,K}$. In particular:

- *Left invariance*: By Equation (18),
  (31) $$D_{C,K}^{\left(\Phi \right)}\left(a,b\right)=D_{C,K}^{\left(\Phi \right)}\left(c*a,c*b\right)$$
  for all a,b,c∈G, hence,
  (32) $$D_{C,K}^{\left(\Phi \right)}\left(a,b\right)=D_{C,K}^{\left(\Phi \right)}\left(e,a^{-1}*b\right)=D_{C,K}^{\left(\Phi \right)}\left(e,b^{-1}*a\right),$$
  where *e* is the identity of G.
- *Norm-based definition*: By Equation (21),
  (33) $$D_{C,K}^{\left(\Phi \right)}\left(a,b\right)={∥\Phi {\left(a\right)}^{-1}\circ \Phi \left(b\right)∥}_{C,K}={∥\Phi {\left(b\right)}^{-1}\circ \Phi \left(a\right)∥}_{C,K},$$
  where ${∥\cdot ∥}_{C,K}$ is the Cayley/Kendall norm in Sym(G), i.e.,
  (34) $${∥r∥}_{C,K}=d_{C,K}\left(e,r\right)$$
  for all r∈Sym(G), e being the identity of Sym(G).

From Equations (16) and (30), it follows

(35) $$D_{C}^{\left(\Phi \right)}\left(a,b\right)\leq D_{K}^{\left(\Phi \right)}\left(a,b\right)$$

for all a,b∈G, since Φ(a),Φ(b)∈Sym(G). Furthermore, by Equation (24),

(36) $$D_{C}^{\left(\Phi \right)}\left(a,b\right)\in \left\{0,1,\ldots ,D_{C,\max}^{\left(\Phi \right)}\left(\left|G\right|\right)\right\},\;\mathrm{where}\;D_{C,\max}^{\left(\Phi \right)}\left(\left|G\right|\right)=\left|G\right|-1,$$

and, by Equation (25),

(37) $$D_{K}^{\left(\Phi \right)}\left(a,b\right)\in \left\{0,1,\ldots ,D_{K,\max}^{\left(\Phi \right)}\left(\left|G\right|\right)\right\},\;\mathrm{where}\;D_{K,\max}^{\left(\Phi \right)}\left(\left|G\right|\right)=\frac{\left|G\right|\left(\left|G\right|-1\right)}{2}.$$

**Remark** **4.** *In the case G=Sym(L) of Section 3.2, the distances $d_{C,k}\left(r,s\right)$ take on all integer values ranging from 0 to their respective maxima $d_{C,\max}=L-1$ (Equation (24)), and $d_{K,\max}=L\left(L-1\right)/2$ (Equation (25)); think of the corresponding adjacency graphs. However, this does not happen with $D_{C,K}^{\left(\Phi \right)}\left(a,b\right)$ because Φ(G) is a subgroup of cardinality $\left|G\right|$ of the group Sym(G), whose cardinality is $\left|G\right|!$, so not all possible distances can be realized (unless $\left|G\right|=2$). We call “forbidden distances for $D_{C,K}^{\left(\Phi \right)}$" the values in $\left\{0,1,\ldots ,D_{C,K,\max}^{\left(\Phi \right)}\right\}$ that are missing in the adjacency subgraph of Φ(G); otherwise, they are called allowed or admissible distances. By Equation (32) (or Remark 2), the admissible distances for $D_{C,K}^{\left(\Phi \right)}$ can be read in the row $\left(D_{C,K}^{\left(\Phi \right)}\left(e,c\right):c\in G)\right)$ of the distance matrix.*

In general, the definition (30) depends on the implementation of Cayley’s isomorphism Φ, e.g., whether Φ(a) is (i) a left translation $\Lambda_{a}$ (Equation (3)), (ii) a right translation $R_{a}$ (Equation (4)), or (iii) an adjoint action (Equation (5)). For simplicity, we mainly use the implementation (i), so that $\Lambda_{a}\left(b\right)$ can be read row-wise in the multiplicaction table of G (see Equation (6)), in which case we write $D_{C,K}^{\left(\Lambda \right)}$ for $D_{C,K}^{\left(\Phi \right)}$. In case (ii), we will write $D_{C,K}^{\left(R\right)}$.

**Example** **5.** *The only non-cyclic group of order 4 is the Klein four-group K, defined by the multiplication table*

(38)

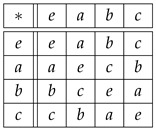


*so that*

(39)

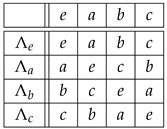


*According to Equations (36) and (37), $D_{C}^{\left(\Lambda \right)}\left(r,s\right)\in \left\{0,1,2,3\right\}$ and $D_{K}^{\left(\Lambda \right)}\left(r,s\right)\in \left\{0,1,\ldots ,6\right\}$. From (39) it follows*

(40)

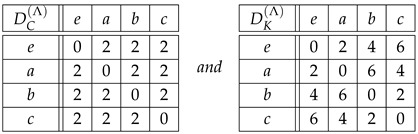


*so the forbidden values of $D_{C}^{\left(\Lambda \right)}\left(r,s\right)$ are {1,3} and the forbidden values of $D_{K}^{\left(\Lambda \right)}\left(r,s\right)$ are {1,3,5}. Note that K is abelian (as any group whose cardinality is the square of a prime number) since the multiplication table in Equation (38) is symmetric and every element other than the identity has order 2, i.e., every element is its own inverse. Therefore,*

$$R_{r}\left(s\right)=s*r^{-1}=s*r=r*s=\Lambda_{r}\left(s\right),$$

*i.e., the isomorphic copies $\Lambda_{r},R_{r}\in \mathrm{Sym}\left(K\right)$ are the same for all r∈K, which implies $D_{C,K}^{\left(R\right)}=D_{C,K}^{\left(\Lambda \right)}$. Labeling the elements e,a,b,c as 1,2,3,4, one can locate the four copies $\left\{\Lambda_{r}:r\in K\right\}$ of the group K in the Kendall adjacency graph of Sym(4), Figure 2, and read there the distances in the right table of Equation (40). For example,*

$$D_{K}^{\left(\Lambda \right)}\left(a,b\right)=d_{K}\left(\Lambda_{a},\Lambda_{b}\right)=d_{K}\left(aecb,bcea\right)=d_{K}\left(2143,3412\right)=6.$$

As a final remark, note that when G=Sym(L), $D_{C,K}^{\left(\Phi \right)}\left(r,s\right)$ does not become $d_{C,K}\left(r,s\right)$, as one might think. The reason is that, in that event, $d_{C,K}\left(r,s\right)$ is defined on Sym(L)×Sym(L), while $D_{C,K}^{\left(\Phi \right)}\left(r,s\right)=d_{C,K}\left(\Phi \left(r\right),\Phi \left(s\right)\right)$, where $d_{C,K}\left(\Phi \left(r\right),\Phi \left(s\right)\right)$ is defined on Sym(Sym(L))×Sym(Sym(L))=Sym(L!)×Sym(L!). In other terms, the definition domain and the range of Cayley’s isomorphism Φ:Sym(L)→Sym(L!) are different also in the particular case G=Sym(L), which prevents Φ from becoming the identity (unless L=2). However, this does not prevent $d_{C,K}\left(r,s\right)$ and $D_{C,K}^{\left(\Lambda \right)}\left(r,s\right)$ from providing the same qualitative and even quantitative information, as shown in Example 6 below and Section 6. This fact supports the consistency of our approach to group metrics based on Cayley’s isomorphism.

**Example** **6.** *Tables (41) and (42) below show the distances $d_{C}\left(\Lambda_{r},\Lambda_{s}\right)=:D_{C}^{\left(\Lambda \right)}\left(r,s\right)$ and $d_{K}\left(\Lambda_{r},\Lambda_{s}\right)=:D_{K}^{\left(\Lambda \right)}\left(r,s\right)$ for r,s∈Sym(3), and $\Lambda_{r}=\Phi \left(r\right)$, $\Lambda_{s}=\Phi \left(s\right)\in \mathrm{Sym}\left(6\right)$, see table (8):*(41)
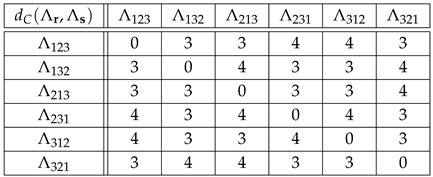
(42)
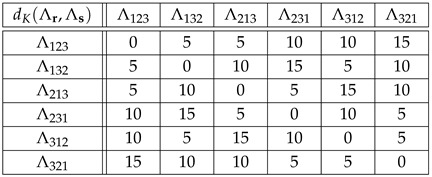
*For instance, if we encode the permutations of Sym(3) as*

(43) 123=1,132=2,213=3,231=4,312=5,321=6,

*then*

$$D_{C}^{\left(\Lambda \right)}\left(213,321\right)=d_{C}\left(\Lambda_{213},\Lambda_{321}\right)=d_{C}\left(341265,654321\right)=4,$$

*while*

$$D_{K}^{\left(\Lambda \right)}\left(213,321\right)=d_{K}\left(\Lambda_{213},\Lambda_{321}\right)=d_{K}\left(341265,654321\right)=10.$$

*Note that if we replace* 3 *by* 1 *and* 4 *by* 2 *in Equation (41) for $d_{C}\left(\Lambda_{r},\Lambda_{s}\right)$, then we obtain Equation (28) for $d_{C}\left(r,s\right)$. Furthermore, if we divide $d_{K}\left(\Lambda_{r},\Lambda_{s}\right)$ in Equation (42) by* 3*, then we obtain Equation (29) for $d_{K}\left(r,s\right)$, i.e.,*

(44) $$D_{K}^{\left(\Lambda \right)}\left(r,s\right)=3d_{K}\left(r,s\right)$$

*for all r,s∈Sym(3). We conclude that the results obtained using $d_{C,K}\left(r,s\right)$ in G=Sym(3) and $D_{C,K}^{\left(\Lambda \right)}\left(r,s\right)$ in Φ(G)⊂Sym(6) are equivalent. According to Equations (41) and (42), the allowed distances for $D_{C}^{\left(\Lambda \right)}$ are {0,3,4} out of {0,1,…,5}, while the allowed distances for $D_{K}^{\left(\Lambda \right)}$ are $\left\{0,5,10,15\right\}=\{5k:0\leq k\leq 3=d_{K,\max}\left(3\right)\}$ out of {0,1,…,15}.*

### 4.2. Distances with Respect to a Generating Set

For the time being, let G be a finite or infinite group. A finite set $S=\{s_{1},\ldots ,s_{n}\}\subset G$ is a *generating set* (or generator) of G if every a∈G can be written as a finite product of elements of *S* and their inverses. In particular, groups generated by a single element are called cyclic. For example, $\left\{\theta^{0},\theta^{1},\ldots ,\theta^{n-1}\right\}$ endowed with $\theta^{i}*\theta^{j}=\theta^{k}$, where k=i+j mod *n* is a cyclic group of order *n* with generator $S=\{\theta^{1}\}$. The (edit) distance (or *word metric*) $d_{S}\left(a,b\right)$ between the elements *a* and *b* of a finitely generated group (in particular of a finite group) G is defined as the minimum number of elements from the generating set *S* needed to transform *a* into *b*. That is, if $b=a*s_{1}*\ldots *s_{k}$, where $s_{i}\in S$ (or $s_{i}^{-1}\in S$), then $d_{S}\left(a,b\right)$ is the smallest possible value of *k*. Therefore, the distance $d_{S}$ depends on the generating set *S*. In particular, if G=Sym(L), then the Cayley distance $d_{C}\left(r,s\right)$ of Section 3.2 is the distance $d_{S}$ with respect to the generating set of all transpositions, while the Kendall distance $d_{K}\left(r,s\right)$ is the distance $d_{S}$ with respect to the generating set of all adjacent transpositions.

**Example** **7.** *For the cyclic group $G=\{\theta^{0},\theta^{1},\theta^{2},\theta^{3}\}$ of Example 2, the distances with respect to the generating set $S=\{\theta^{1}\}$ are the following:*

(45)

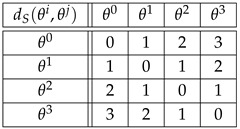


*As for the distances $D_{K}^{\left(\Lambda \right)}\left(\theta^{i},\theta^{j}\right)=d_{K}\left(\Lambda_{\theta^{i}},\Lambda_{\theta^{j}}\right)$, we find (see Equation (10)):*

(46)

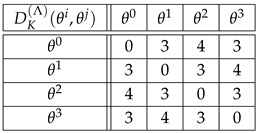


*For example,*

(47) $$D_{K}^{\left(\Lambda \right)}\left(\theta^{2},\theta^{3}\right)=d_{K}\left(\Lambda_{\theta^{2}},\Lambda_{\theta^{3}}\right)=d_{K}\left(\theta^{2}\theta^{3}\theta^{0}\theta^{1},\theta^{3}\theta^{0}\theta^{1}\theta^{2}\right)=3.$$

*If right translations (4) are used instead of left translations (3), then $D_{K}^{\left(R\right)}\left(\theta^{i},\theta^{j}\right)=d_{K}\left(R_{\theta^{i}},R_{\theta^{j}}\right)$ happens to be the same as in Equation (46). For example,*

(48) $$D_{K}^{\left(R\right)}\left(\theta^{2},\theta^{3}\right)=d_{K}\left(R_{\theta^{2}},R_{\theta^{3}}\right)=d_{K}\left(\theta^{2}\theta^{3}\theta^{0}\theta^{1},\theta^{1}\theta^{2}\theta^{3}\theta^{0}\right)=3.$$

*If the group elements $\theta^{0},\theta^{1},\theta^{2},\theta^{3}$ are labeled 1,2,3,4, respectively, then the above distances can be read in the Kendall adjacency graph of Sym(4), Figure 2. For example, distances (47) and (48) read $d_{K}\left(3412,4123\right)$ and $d_{K}\left(3412,2341\right)$, respectively.*

### 4.3. Discussion

When comparing the distances $D_{C,K}^{\left(\Phi \right)}\left(a,b\right)$ and $d_{S}\left(a,b\right)$ for finite groups, a possible advantage of the former is its expediency, in the sense that $D_{C,K}^{\left(\Phi \right)}$ dispenses with generating sets and, hence, with the search for minimal descriptions of *b* as products of the form $a*s_{1}*\ldots *s_{k}$. In addition, there are algorithms (such as the bubble-sort algorithm) that compute $D_{C}^{\left(\Phi \right)}$ in time $O(\left|G\right|)$ and $D_{K}^{\left(\Phi \right)}$ in time $\;O(\left|G\right|\log\left|G\right|)$ [28]. Computational issues are briefly discussed in Section 6.

On the other hand, a possible shortcoming of the distances $D_{C,K}^{\left(\Phi \right)}$ in applications is the existence of forbidden values pointed out in Remark 4. For instance, the presence of such gaps in the distances between the algebraic representations of two coupled time series (see Section 5) might be misinterpreted as a dynamical characteristic of the underlying systems, e.g., full or generalized synchronization. So, the forbidden values for $D_{C,K}^{\left(\Phi \right)}$ must be identified in advance, which can be easily done by calculating the row $\left(D_{C,K}^{\left(\Phi \right)}\left(e,c\right):c\in G)\right)$ of the distance matrix (Remark 4). Alternatively, they can be identified using independent white noises. We come back to this point in Section 6.

In sum, when embedding a group G in $\mathrm{Sym}(\left|G\right|)$ via Cayley’s isomorphism Φ, we are encoding the $\left|G\right|$ elements $\{a_{1},\ldots ,a_{\left|G\right|}\}\in G$ as the $\left|G\right|$ permutations $\Phi \left(a\right)=\left(b_{1},b_{2},\ldots ,b_{\left|G\right|}\right)$, where $\left(b_{1},b_{2},\ldots ,b_{\left|G\right|}\right)$ is a shuffle of $\left(a_{1},\ldots ,a_{\left|G\right|}\right)$; see Equation (6) for Φ being the left translation $a_{i}\mapsto \Lambda_{a_{i}}$. The penalty for doing so is a more complex representation of the elements of G. The pay-off is a general and computationally efficient metric $D_{C,K}^{\left(\Phi \right)}$. In principle, there may be symmetric groups Sym(M) with $M<\left|G\right|$ in which G can be embedded, but finding such symmetric groups, in particular, the minimum-order one, is rather difficult in general [29,30]. In any case, note that in the practice of symbolic representation of time series, the alphabets used have low cardinality.

## 5. Distances for Group-Valued Time Series and Algebraic Representations

In this section, we explore possible applications of permutation-based distances to group-valued time series. Examples of group-valued time series include binary and *n*-ary time series. In the first case, G={0,1}, endowed with the XOR operation (addition modulo 2); these time series arise in digital communications and cryptography. The second example is a generalization, also used in digital communications: G={0,1,…,n−1} endowed with addition modulo *n*.

The perhaps most familiar example of group-valued time series is the ordinal representation of real-value time series, introduced in Section 3.1. A generalization thereof is the concept of algebraic representation.

**Definition** **7.** *We say that a symbolic representation $\alpha ={\left(a_{t}\right)}_{t\geq 0}$ of a time series is an algebraic time series if its elements $a_{t}$ belong to a finite group (G,∗).*

Since here we are interested in practical applications, consider two finite G-valued time series $\alpha ={\left(a_{t}\right)}_{1\leq t\leq N}$ and $\beta ={\left(b_{t}\right)}_{1\leq t\leq N}$ of length *N*. In time series analysis, α and β could be ordinal representations of two coupled real-valued time series ${\left(x_{t}\right)}_{1\leq t\leq N}$ and ${\left(y_{t}\right)}_{1\leq t\leq N}$, respectively. To carry out a data-driven analysis of the coupled dynamics of the underlying systems (think of various types of synchronization), or to measure the similarity between α and β, there are a number of metrics that we review in Section 5.1. In Section 5.2, we discuss how to extract information with those metrics.

### 5.1. String Metrics for Group-Valued Time Series

Below, we mention perhaps the most common metrics. Each of them targets specific situations.

**(i)** 
Some of the metrics to quantify the similarity of two symbolic time series such as α and β are based on the probability distributions of their symbols (estimated by their frequencies) [31]. This category includes the Kullback–Leibler (KL) divergence (usually symmetrized via an arithmetic or harmonic mean) [8], the Jensen–Shannon (JS) divergence [32], the JS distance (which is the square root of the JS divergence) [33], the permutation JS distance [34,35], the Hellinger distance [36], the Wasserstein distance [37,38], the total variation distance [39,40] and more. Since in this paper we are interested in harnessing the algebraic structure of the symbolic data (if any), we will dispense with entropic distances.
**(ii)** 
One can also exploit the algebraic structure of G and calculate the transcription of α and β [8], that is, the time series $\tau ={\left(\tau_{t}\right)}_{t\geq 0}$, where $\tau_{t}=b_{t}*a_{t}^{-1}$ (right translations by $a_{t}$) or $\tau_{t}=a_{t}^{-1}*b_{t}$ (left translations by $a_{t}^{-1}$), see Equations (4) and (3). Trancriptions of coupled time series in an ordinal representation have been used to study different aspects of coupled dynamics: complexity [8,41], synchronization [8,41], information directionality (or causality) [42], features for classification [43], etc. Interestingly, if G=Sym(L), then the distance between the ordinal patterns $a_{t}$ and $b_{t}$ can be written as the norm ${∥\cdot ∥}_{C,K}$ of the transcript $a_{t}^{-1}\circ b_{t}$, see Equation (21). Otherwise, we embed G into Sym(G) via Cayley’s isomorphism Φ:G→Sym(G) and, again, the distance between the ordinal patterns $\Phi (a_{t})$ and $\Phi (b_{t})$ can be written as the norm ${∥\cdot ∥}_{C,K}$ of the transcript $\Phi {\left(a_{t}\right)}^{-1}\circ \Phi \left(b_{t}\right)$, see Equation (33).
**(iii)** 
Since a window $a_{t}^{W}:=a_{t},a_{t+1},\ldots ,a_{t+W-1}$ of size *W* of any G-valued time series $\alpha ={\left(a_{t}\right)}_{t\geq 0}$ can be viewed as a string of symbols of length *W*, we can borrow a number of string metrics from information theory, computer science and computational linguistics to compare $a_{t}^{W}$ and $b_{t}^{W}:=b_{t},b_{t+1},\ldots ,b_{t+W-1}$, where (unlike permutations) these strings can have repeated symbols. Thus, the *Hamming distance* between two strings of equal length is the number of positions at which the corresponding symbols differ [44]. The *Damerau–Levenshtein distance* considers insertions, deletions, substitutions and adjacent transpositions of symbols [45,46,47]. Such metrics are also examples of edit distances. Finally, we also mention the *Jaro–Winkler similarity coefficient* (not a true distance) which, like the Hamming distance, is based on symbol matching [48,49].

### 5.2. Extracting Information with $d_{C,K}$ and $D_{C,K}^{\left(\Phi \right)}$

Next, we focus on the distances $d_{C,K}$ for the group Sym(L) (Section 3.2) and $D_{C,K}^{\left(\Phi \right)}$ for other groups (Section 3.2) and their applications to the analysis of G-valued time series and algebraic representations. The idea is to measure the distance between (A) simultaneous symbols $a_{t}$ and $b_{t}$, or (B) concurrent windows $a_{t}^{W}$ and $b_{t}^{W}$, and thereby characterize the similarity or dissimilarity of the symbolic time series α and β. To this end, we consider sliding windows $a_{t}^{W}$ and $b_{t}^{W}$, 1≤t≤N−W+1, with the same size W≥1, where we allow W=1 in order to include distances between simultaneous symbols.

**CASE A:**  W=1. To unify the notation, we will write $\mathrm{dist}(a_{t},b_{t})$ for the distance between the elements $a_{t},b_{t}\in G$, with the understanding that $\mathrm{dist}\left(a_{t},b_{t}\right)=d_{C,K}\left(a_{t},b_{t}\right)$ if G=Sym(L) and $\mathrm{dist}\left(a_{t},b_{t}\right)=D_{C,K}^{\left(\Phi \right)}\left(a_{t},b_{t}\right)$ otherwise. Therefore,
  (49) $$\mathrm{dist}\left(a_{t},b_{t}\right)\in \left\{0,1,\ldots ,{\mathrm{dist}}_{\max}\right\},$$
  where
  (50) $${\mathrm{dist}}_{\max}\left\{\begin{array}{cc} =L-1 & \mathrm{if}\;\mathrm{dist}=d_{C}\left(\mathrm{Equation}\left(24\right)\right),\\ =L(L-1)/2 & \mathrm{if}\;\mathrm{dist}=d_{K}\left(\mathrm{Equation}\left(25\right)\right),\\ \leq \left|G\right|-1 & \;\;\mathrm{if}\;\mathrm{dist}=D_{C}^{\left(\Phi \right)}\left(\mathrm{Equation}\left(36\right)\right),\\ \leq \left|G\right|\left(\left|G\right|-1\right)/2 & \;\;\mathrm{if}\;\mathrm{dist}=D_{K}^{\left(\Phi \right)}\left(\mathrm{Equation}\left(37\right)\right). \end{array}\right.$$
  where the inequalities in Equation (50) allow for the possibility that $D_{C,K,\max}^{\left(\Phi \right)}$ is a forbidden distance (Remark 4). As a result of calculating $\mathrm{dist}(a_{t},b_{t})$ for 1≤t≤N, we obtain the integer-valued time series
  (51) $${\left(\mathrm{dist}\left(a_{t},b_{t}\right)\right)}_{1\leq t\leq N}.$$
  According to Equation (50), $d_{C,\max}<d_{K,\max}$ and $D_{C,\max}^{\left(\Phi \right)}<D_{K,\max}^{\left(\Phi \right)}$, except for L=2. Therefore, $d_{K}$ and $D_{K}^{\left(\Phi \right)}$ have greater differentiating power in applications than their Cayley counterparts due to their larger ranges.
**CASE B:**  W>1. Consider now the windows $a_{t}^{W}=\left(a_{t},a_{t+1},\ldots ,a_{t+W-1}\right)$ and $b_{t}^{W}=\left(b_{t},b_{t+1},\ldots ,b_{t+W-1}\right)$ as *W*-dimensional vectors in the corresponding Cartesian product of the metric space (G,dist). In this case, we have the whole family of $l_{p}$ distances, p≥1, at our disposal. Well-known instances include the so-called *Manhattan distance*,
  (52) $${\mathrm{dist}}_{1}\left(a_{t}^{W},b_{t}^{W}\right)=\sum_{k=0}^{W-1}\mathrm{dist}\left(a_{t+k},b_{t+k}\right),$$
  the *Euclidean distance*,
  (53) $${\mathrm{dist}}_{2}\left(a_{t}^{W},b_{t}^{W}\right)={\left(\sum_{k=0}^{W-1}\mathrm{dist}{\left(a_{t+k},b_{t+k}\right)}^{2}\right)}^{1/2},$$
  and the *Chebychev distance*,
  (54) $${\mathrm{dist}}_{\infty }\left(a_{t}^{W},b_{t}^{W}\right)=\max\left\{\mathrm{dist}(a_{t+k},b_{t+k}):0\leq k\leq W-1\right\}.$$
  As a result, we obtain the time series
  (55) $${\left({\mathrm{dist}}_{p}\left(a_{t}^{W},b_{t}^{W}\right)\right)}_{1\leq t\leq N-W+1}$$
  which is integer-valued for =1,∞, and real-valued otherwise.

Once the metric information from the G-valued time series α and β has been collected element-wise (51) and/or window-wise (55), one can proceed in several ways to process the information. We discuss some simple ways in Section 6.

## 6. Numerical Simulations

**Figure 3:**
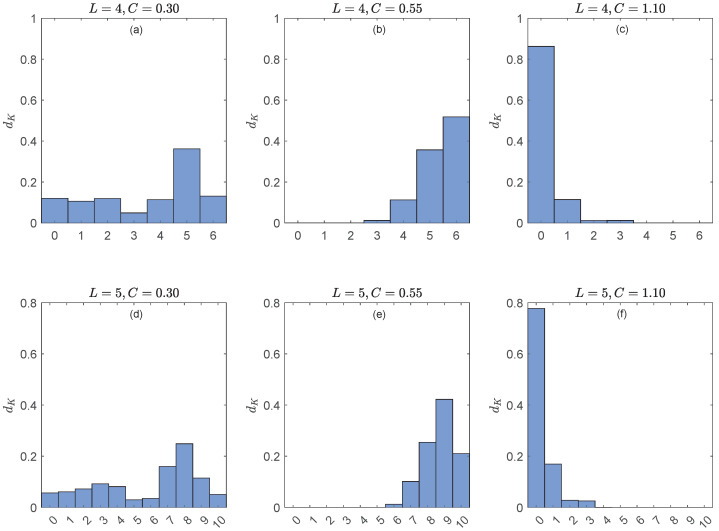
**Top row**: Probability distributions of the Kendall distances $d_{K}\left(r_{t},s_{t}\right)$ for the algebraic representation of the time series *x* and *y* with the group G=Sym(4) (i.e., ordinal patterns of length L=4) and coupling strengths C=0.30 (**left panel**), 0.55 (**middle panel**) and 1.10 (**right panel**). **Bottom row**: Same as top row for the representation group G=Sym(5) (i.e., ordinal patterns of length L=5).

In this section, we illustrate the application of permutation-based distances to algebraic representations with numerical simulations. To this end, we revisit a model composed of two unidirectionally coupled, non-identical Henon systems, used in [50] to study generalized synchronization. The equations of the driver *X* are

(56) $$\left\{\begin{array}{c} x_{t+1}^{\left(1\right)}=1.4-{\left(x_{t}^{\left(1\right)}\right)}^{2}+0.1x_{t}^{\left(2\right)}\\ x_{t+1}^{\left(2\right)}=x_{t}^{\left(1\right)} \end{array}\right.$$

and the equations of the responder *Y* are

(57) $$\left\{\begin{array}{c} y_{t+1}^{\left(1\right)}=1.4-\left[Cx_{t}^{\left(1\right)}y_{t}^{\left(1\right)}+\left(1-C\right){\left(y_{t}^{\left(1\right)}\right)}^{2}\right]+0.3y_{t}^{\left(2\right)}\\ y_{t+1}^{\left(2\right)}=y_{t}^{\left(1\right)} \end{array}\right.$$

where C≥0 is the *coupling strength*. It is numerically proved in [50] that this system has *generalized synchronization* for *C* in a small interval around 0.55 and for C≳1 ([50], [Figure 3]).

For a given coupling strength *C*, let $x={\left(x_{t}^{\left(1\right)}\right)}_{1\leq t\leq 10000}$ and $y={\left(y_{t}^{\left(1\right)}\right)}_{1\leq t\leq 10000}$ be two stationary time series of length N=10000 composed of the first components of the states $x_{t}=\left(x_{t}^{\left(1\right)},x_{t}^{\left(2\right)}\right)$ of the driver and $y_{t}=\left(y_{t}^{\left(1\right)},y_{t}^{\left(2\right)}\right)$ of the responder, respectively, and generated with seeds $x_{0}=\left(0,0.9\right)$ and $y_{0}=\left(0.75,0\right)$ (after discarding the initial transient). Let $\alpha ={\left(r_{t}\right)}_{1\leq t\leq 10000-L+1}$ and $\beta ={\left(s_{t}\right)}_{1\leq t\leq 10000-L+1}$ be the algebraic representations of *x* and *y* with ordinal patterns of length 3≤L≤6. The values chosen for the coupling strength are C=0.30,0.55,1.10.

Next we computed different types of distances between α and β from those presented in Section 5.2. Here, we present only the results with the Kendall distances $d_{K}\left(r_{t},s_{t}\right)$ and $D_{K}^{\left(\Lambda \right)}\left(r_{t},s_{t}\right)$ because, as explained there, they have greater differentiating power than $d_{C}$ and $D_{C}^{\left(\Lambda \right)}$. As for the distances ${\mathrm{dist}}_{p}\left(r_{t}^{W},s_{t}^{W}\right)$, we used p=1,2,∞ (Equations (52)–(54)). Irrational values of ${\mathrm{dist}}_{2}\left(r_{t}^{W},s_{t}^{W}\right)$ were rounded to the integer *n* if ${\mathrm{dist}}_{2}\left(r_{t}^{W},s_{t}^{W}\right)\in \left(n-0.5,\;n+0.5\right]$. To facilitate analysis, we transformed the data ${\left(d_{K}\left(r_{t},s_{t}\right)\right)}_{1\leq t\leq N-L+1}$, ${\left(D_{K}^{\left(\Lambda \right)}\left(r_{t},s_{t}\right)\right)}_{1\leq t\leq N-L+1}$ and ${\left({\mathrm{dist}}_{p}\left(r_{t}^{W},s_{t}^{W}\right)\right)}_{1\leq t\leq N-L-W+2}$ into (empirical) probability distributions for the distance values.

Figure 3 illustrates CASE A of Section 5.2, i.e., W=1. Here, G=Sym(4) (top row) and G=Sym(5) (bottom row). The main conclusions can be summarized as follows.

- For C=0.30 (no synchronization, panels (a) and (d)), all possible values {0,1,…,L(L−1)/2} of $d_{K}$ are realized.
- For C=0.55 (“weak synchronization”, panels (b) and (e)), only the greater values of $d_{K}$ are allowed.
- For C=1.10 (“strong synchronization”, panels (c) and (f)), only the smaller values of $d_{K}$ are allowed.
- So, $d_{K}$ detects that the generalized synchronizations at C=0.55 and C=1.10 are different: the former forbids the shorter distances between simultaneous ordinal patterns $r_{t}$ and $s_{t}$, while the latter forbids large distances.
- The results for each *C* are consistently similar.

We conclude that the distance $d_{K}$ is sensitive to dynamical changes in coupled systems and robust with respect to the length of the ordinal patterns.

**Figure 4:**
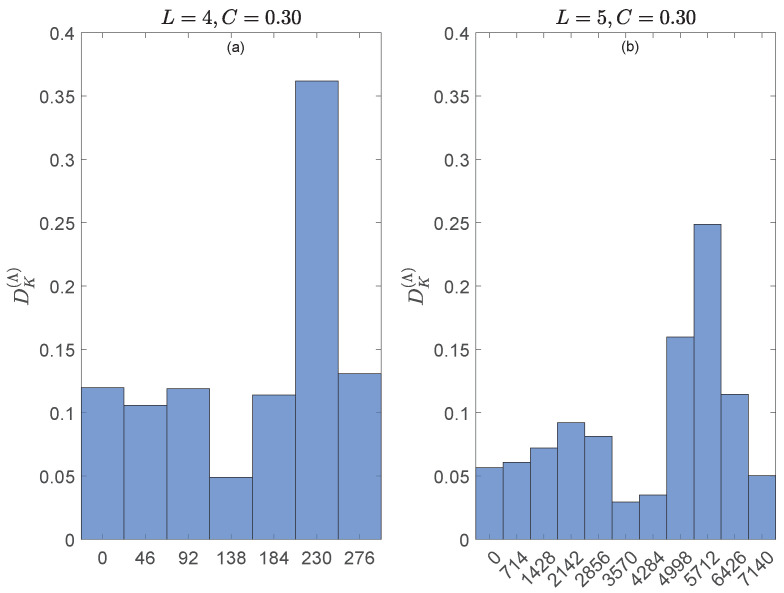
Probability distributions of the allowed distances for $D_{K}^{\left(\Lambda \right)}\left(r_{t},s_{t}\right)$ for the algebraic representation of the time series *x* and *y* with the group G=Sym(4) (panel (**a**)) and G=Sym(5) (panel (**b**)). C=0.30 in both panels so that all L(L−1)/2+1 allowed distances for $D_{K}^{\left(\Lambda \right)}\left(r_{t},s_{t}\right)$ (listed along the horizontal axes) are actually realized.

At this point, we draw on Figure 3 to, as in Example 6, check the consistency of the results obtained with $d_{K}$ and $D_{K}^{\left(\Lambda \right)}$, this time using G=Sym(4) and G=Sym(5). Figure 4 shows the probability distribution of the allowed distances for $D_{K}^{\left(\Lambda \right)}\left(r_{t},s_{t}\right)$ with L=4 (panel (a)) and L=5 (panel (b)). The coupling strength in both panels is C=0.30, so that all L(L−1)/2+1 allowed distances are realized. The allowed distances for $D_{K}^{\left(\Lambda \right)}\left(r_{t},s_{t}\right)$, listed along the horizontal axes in Figure 4, happen to be $\left\{46k:0\leq k\leq 6=d_{K,\max}\left(4\right)\right\}$ for L=4 and $\left\{714k:0\leq k\leq 10=d_{K,\max}\left(5\right)\right\}$ for L=5. Comparison of panels (a) and (b) of Figure 4 with panels (a) and (d) of Figure 3, respectively, shows that the probability distributions of $D_{K}^{\left(\Lambda \right)}\left(r_{t},s_{t}\right)$ and $d_{K}\left(r_{t},s_{t}\right)$ are exactly the same for L=4,5 and C=0.30, except for the labeling of the distances; notice the change of scales. In fact, and similarly to Equation (44), numerical calculations show that (i)

(58) $$D_{K}^{\left(\Lambda \right)}\left(r,s\right)=46d_{K}\left(r,s\right)$$

for all r,s∈Sym(4), where $46=\min\{d_{K}\left(\Lambda_{r},\Lambda_{s}\right)>0:r,s\in \mathrm{Sym}\left(4\right)\}$, and (ii)

(59) $$D_{K}^{\left(\Lambda \right)}\left(r,s\right)=714d_{K}\left(r,s\right)$$

for all r,s∈Sym(5), where $714=\min\{d_{K}\left(\Lambda_{r},\Lambda_{s}\right)>0:r,s\in \mathrm{Sym}\left(5\right)\}$. For example, $d_{K}\left(\Lambda_{1234},\Lambda_{1243}\right)=46$ and $d_{K}\left(\Lambda_{12345},\Lambda_{12354}\right)=714$. The same occurs for C=0.55 and C=1.10 (not shown).

**Figure 5:**
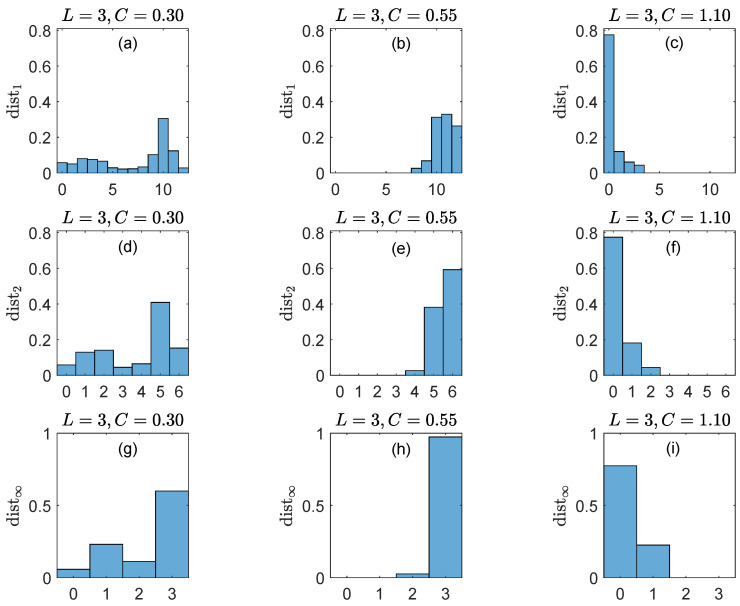
**Top row**: Probability distributions of the distance ${\mathrm{dist}}_{1}\left(r_{t}^{4},s_{t}^{4}\right)$ for the algebraic representation of the time series *x* and *y* with the group G=Sym(3) (ordinal patterns of length L=3) and coupling strengths C=0.30 (**left panel**), 0.55 (**middle panel**) and 1.10 (**right panel**). **Middle row**: Same as top row for the distance ${\mathrm{dist}}_{2}\left(r_{t}^{4},s_{t}^{4}\right)$. **Bottom row**: Same as top row for the distance ${\mathrm{dist}}_{\infty }\left(r_{t}^{4},s_{t}^{4}\right)$.

Figure 5 illustrates CASE B of Section 5.2, i.e., W>1. Here, W=4 with G=Sym(3), $\mathrm{dist}\left(r_{t},s_{t}\right)=d_{K}\left(r_{t},s_{t}\right)$, and the distance ${\mathrm{dist}}_{p}\left(r_{t}^{4},s_{t}^{4}\right)$ is (i) ${\mathrm{dist}}_{1}\left(r_{t}^{4},s_{t}^{4}\right)\in \left\{0,1,\ldots ,12\right\}$ in the top row, (ii) ${\mathrm{dist}}_{2}\left(r_{t}^{4},s_{t}^{4}\right)\in \left[0,6\right]$ in the middle row and (iii) ${\mathrm{dist}}_{\infty }\left(r_{t}^{4},s_{t}^{4}\right)\in \left\{0,1,2,3\right\}$ in the bottom row. The main conclusions can be summarized as follows.

- Due to the monotony property of the *p*-norms (${∥\cdot ∥}_{p}\geq {∥\cdot ∥}_{p^{\prime }}$ for 1≤p≤$p^{\prime }\leq \infty$), the distances with smaller parameters *p* (${\mathrm{dist}}_{1}$ and ${\mathrm{dist}}_{2}$ in Figure 3) have greater differentiating power.
- The results shown in Figure 3 (obtained with sliding windows of 4 consecutive ordinal patterns of length 3) are qualitatively similar to the results shown in Figure 1 (obtained with simultaneous pairs $\left(r_{t},s_{t}\right)$ of ordinal patterns of lengths 4 and 5).

We conclude that the distances ${\mathrm{dist}}_{p}$ are also sensitive to dynamical changes in coupled systems and robust with respect to the parameter p≥1.

To wrap up the previous discussion, we are also going to compare the computational times of $D_{C,K}^{\left(\Lambda \right)}\left(a_{t},b_{t}\right)$ (Section 4.1) and $d_{S}\left(a_{t},b_{t}\right)$ (Section 4.2), where ${\left(a_{t}\right)}_{1\leq t\leq N}$, ${\left(b_{t}\right)}_{1\leq t\leq N}$ are G-valued time series. Rather than using ad hoc groups and coupled time series, we take advange of the above ordinal representations α and β, and benchmark the computational cost of computing $D_{C,K}^{\left(\Lambda \right)}\left(r_{t},s_{t}\right)$ for G=Sym(L), 3≤L≤6 (the usual ordinal pattern lengths in applications), N= 10,000 and C=0.30, against the computational cost of calculating $d_{K}\left(r_{t},s_{t}\right)$ for the same group and settings. We choose C=0.30 so that all allowed ordinal patterns are realized (see Figure 3 and Figure 4). Table 1 shows the times in seconds of the corresponding calculations with a laptop (Intel I9 processor, 8 cores, 64 GB of RAM, 8 GB of GPU memory) and a non-paralellized algorithm.

Altogether, the above numerical results support the usefulness of distances $d_{C,K}$, $D_{C,K}^{\left(\Phi \right)}$ and ${\mathrm{dist}}_{p}$ in the analysis of group-valued time series.

## 7. Conclusions

The results presented in this paper are an outgrowth of the study of transcripts and their applications to time series analysis in algebraic representations (Section 5), which are a generalization of transcripts in ordinal representations [8]. Indeed, the concept of transcript from a group element a∈G to another b∈G or, for that matter, the right translation of *b* by *a* (Equation (4)) leads directly to the isomorphism $\Phi :a\mapsto R\left(a,\cdot \right)=:R_{a}$ from G to a subgroup of the symmetric group Sym(G) (Cayley’s theorem). In turn, the elements of Sym(G) can be written as numerical or symbolic strings, which allows us to endow Sym(G) with any convenient edit distance, e.g., the Cayley distance $d_{C}$ or the Kendall distance $d_{K}$ of Section 3. This being the case, the isomorphism Φ can be used to transport the distance $d_{C,K}$ in Sym(G) to G, as we did in Section 4. The result is the ordinal pattern-based distance for groups $D_{C,K}^{\left(\Phi \right)}\left(a,b\right)$ proposed in Definition 6.

Metric properties of finite groups is an unsual tool in time series analysis in algebraic representations. Even in the ordinal representation, distances or similarities between time series are usually measured with functionals of probability distributions such as divergences or functions thereof. There are also distances defined in the groups themselves, based on generating sets, which were the subject of Section 4.2. Actually, the distances $d_{C}$ and $d_{K}$ in the permutations groups, discussed in Section 3, are examples of distances with respect to generating sets. A possible advantage of the ordinal pattern-based distance proposed in this paper for any group G is its simplicity and generality, since it dispenses with generating sets and minimal descriptions of elements via generators. Furthermore, there are general-purpose algorithms to efficiently calculate the distances $d_{C}$ and $d_{K}$ in Sym(G) for the low and moderate group cardinalities used in practice, see Table 1.

In the previous sections we have presented the mathematical underpinnings of our approach, which include group actions, Cayley’s theorem, and group representations, as well as its practical implementation. It is remarkable that Cayley’s theorem gives permutations (or ordinal patterns) a certain universality in algebraic representations of time series, although other choices or isomorphisms can be more convenient in practice. For example, the Klein group (Example 5) is isomorphic to $Z_{2}\times Z_{2}$ endowed with XOR addition and the cyclic group $\left\{\theta^{0},\theta^{1},\ldots ,\theta^{n-1}\right\}$ endowed with $\theta^{i}*\theta^{j}=\theta^{k}$, where k=i+j mod *n*, is isomorphic to {0,1,…,n−1} equipped with addition modulo *n*. Some of these groups were used in the previous sections to illustrate the theory. In contrast to the specificities of each group, the group distance introduced in Definition 6 is completely general, since the only input it needs is the multiplication table of the group, and can be efficiently computed. Possible applications were only touched upon in Section 5 because they are the subject of ongoing research. The numerical simulations in Section 6 show the potential of the metric tools discussed in this paper in the analysis of group-valued time series.

## Figures and Tables

**Table 1:** Computation time in seconds of $d_{K}\left(r_{t},s_{t}\right)$ and $D_{C,K}^{\left(\Lambda \right)}\left(r_{t},s_{t}\right)$, 1≤t≤ 10,000.

G	Φ(G)	$d_{K}\left(r_{t},s_{t}\right)$	$D_{C,K}^{\left(\Lambda \right)}\left(r_{t},s_{t}\right)$
Sym(3)	Sym(6)	0.009 s	0.022 s
Sym(4)	Sym(24)	0.010 s	0.038 s
Sym(5)	Sym(120)	0.011 s	0.143 s
Sym(6)	Sym(720)	0.012 s	2.837 s

## Data Availability

The numerical data supporting the conclusions of this article will be made available by the authors on request.

## Appendix A. Cayley and Kendall Distances for the Group Sym (4) (Example 4)

**Table A1:** Distance $d_{C}$ for Sym(4).

$d_{C}$	1234	1243	1324	1342	1423	1432	2134	2143	2314	2341	2413	2431	3124	3142	3214	3241	3412	3421	4123	4132	4213	4231	4312	4321
1234	0	1	1	2	2	1	1	2	2	3	3	2	2	3	1	2	2	3	3	2	2	1	3	2
1243	1	0	2	1	1	2	2	1	3	2	2	3	3	2	2	1	3	2	2	3	1	2	2	3
1324	1	2	0	1	1	2	2	3	1	2	2	3	1	2	2	3	3	2	2	3	3	2	2	1
1342	2	1	1	0	2	1	3	2	2	1	3	2	2	1	3	2	2	3	3	2	2	3	1	2
1423	2	1	1	2	0	1	3	2	2	3	1	2	2	3	3	2	2	1	1	2	2	3	3	2
1432	1	2	2	1	1	0	2	3	3	2	2	1	3	2	2	3	1	2	2	1	3	2	2	3
2134	1	2	2	3	3	2	0	1	1	2	2	1	1	2	2	3	3	2	2	1	3	2	2	3
2143	2	1	3	2	2	3	1	0	2	1	1	2	2	1	3	2	2	3	1	2	2	3	3	2
2314	2	3	1	2	2	3	1	2	0	1	1	2	2	3	1	2	2	3	3	2	2	3	1	2
2341	3	2	2	1	3	2	2	1	1	0	2	1	3	2	2	1	3	2	2	3	3	2	2	1
2413	3	2	2	3	1	2	2	1	1	2	0	1	3	2	2	3	1	2	2	3	1	2	2	3
2431	2	3	3	2	2	1	1	2	2	1	1	0	2	3	3	2	2	1	3	2	2	1	3	2
3124	2	3	1	2	2	3	1	2	2	3	3	2	0	1	1	2	2	1	1	2	2	3	3	2
3142	3	2	2	1	3	2	2	1	3	2	2	3	1	0	2	1	1	2	2	1	3	2	2	3
3214	1	2	2	3	3	2	2	3	1	2	2	3	1	2	0	1	1	2	2	3	1	2	2	3
3241	2	1	3	2	2	3	3	2	2	1	3	2	2	1	1	0	2	1	3	2	2	1	3	2
3412	2	3	3	2	2	1	3	2	2	3	1	2	2	1	1	2	0	1	3	2	2	3	1	2
3421	3	2	2	3	1	2	2	3	3	2	2	1	1	2	2	1	1	0	2	3	3	2	2	1
4123	3	2	2	3	1	2	2	1	3	2	2	3	1	2	2	3	3	2	0	1	1	2	2	1
4132	2	3	3	2	2	1	1	2	2	3	3	2	2	1	3	2	2	3	1	0	2	1	1	2
4213	2	1	3	2	2	3	3	2	2	3	1	2	2	3	1	2	2	3	1	2	0	1	1	2
4231	1	2	2	3	3	2	2	3	3	2	2	1	3	2	2	1	3	2	2	1	1	0	2	1
4312	3	2	2	1	3	2	2	3	1	2	2	3	3	2	2	3	1	2	2	1	1	2	0	1
4321	2	3	1	2	2	3	3	2	2	1	3	2	2	3	3	2	2	1	1	2	2	1	1	0

**Table A2:** Distance $d_{K}$ for Sym(4).

$d_{K}$	1234	1243	1324	1342	1423	1432	2134	2143	2314	2341	2413	2431	3124	3142	3214	3241	3412	3421	4123	4132	4213	4231	4312	4321
1234	0	1	1	2	2	3	1	2	2	3	3	4	2	3	3	4	4	5	3	4	4	5	5	6
1243	1	0	2	3	1	2	2	1	3	4	2	3	3	4	4	5	5	6	2	3	3	4	4	5
1324	1	2	0	1	3	2	2	3	3	4	4	5	1	2	2	3	3	4	4	3	5	6	4	5
1342	2	3	1	0	2	1	3	4	4	5	5	6	2	1	3	4	2	3	3	2	4	5	3	4
1423	2	1	3	2	0	1	3	2	4	5	3	4	4	3	5	6	4	5	1	2	2	3	3	4
1432	3	2	2	1	1	0	4	3	5	6	4	5	3	2	4	5	3	4	2	1	3	4	2	3
2134	1	2	2	3	3	4	0	1	1	2	2	3	3	4	2	3	5	4	4	5	3	4	6	5
2143	2	1	3	4	2	3	1	0	2	3	1	2	4	5	3	4	6	5	3	4	2	3	5	4
2314	2	3	3	4	4	5	1	2	0	1	3	2	2	3	1	2	4	3	5	6	4	3	5	4
2341	3	4	4	5	5	6	2	3	1	0	2	1	3	4	2	1	3	2	4	5	3	2	4	3
2413	3	2	4	5	3	4	2	1	3	2	0	1	5	6	4	3	5	4	2	3	1	2	4	3
2431	4	3	5	6	4	5	3	2	2	1	1	0	4	5	3	2	4	3	3	4	2	1	3	2
3124	2	3	1	2	4	3	3	4	2	3	5	4	0	1	1	2	2	3	5	4	6	5	3	4
3142	3	4	2	1	3	2	4	5	3	4	6	5	1	0	2	3	1	2	4	3	5	4	2	3
3214	3	4	2	3	5	4	2	3	1	2	4	3	1	2	0	1	3	2	6	5	5	4	4	3
3241	4	5	3	4	6	5	3	4	2	1	3	2	2	3	1	0	2	1	5	4	4	3	3	2
3412	4	5	3	2	4	3	5	6	4	3	5	4	2	1	3	2	0	1	3	2	4	3	1	2
3421	5	6	4	3	5	4	4	5	3	2	4	3	3	2	2	1	1	0	4	3	3	2	2	1
4123	3	2	4	3	1	2	4	3	5	4	2	3	5	4	6	5	3	4	0	1	1	2	2	3
4132	4	3	3	2	2	1	5	4	6	5	3	4	4	3	5	4	2	3	1	0	2	3	1	2
4213	4	3	5	4	2	3	3	2	4	3	1	2	6	5	5	4	4	3	1	2	0	1	3	2
4231	5	4	6	5	3	4	4	3	3	2	2	1	5	4	4	3	3	2	2	3	1	0	2	1
4312	5	4	4	3	3	2	6	5	5	4	4	3	3	2	4	3	1	2	2	1	3	2	0	1
4321	6	5	5	4	4	3	5	4	4	3	3	2	4	3	3	2	2	1	3	2	2	1	1	0
